# Bilateral vestibulopathy and age: experimental considerations for testing dynamic visual acuity on a treadmill

**DOI:** 10.1007/s00415-020-10249-z

**Published:** 2020-10-28

**Authors:** D. Starkov, M. Snelders, F. Lucieer, A. M. L. Janssen, M. Pleshkov, H. Kingma, V. van Rompaey, N. Herssens, A. Hallemans, L. Vereeck, C. McCrum, K. Meijer, N. Guinand, A. Perez-Fornos, R. van de Berg

**Affiliations:** 1 Division of Balance Disorders, Department of Otorhinolaryngology and Head and Neck Surgery, Maastricht University Medical Centre, Maastricht, The Netherlands; 2 Faculty of Physics, Tomsk State Research University, Tomsk, Russia; 3 Department of Methodology and Statistics, Care and Public Health Research Institute (CAPHRI), Maastricht University, Maastricht, The Netherlands; 4 Faculty of Medicine and Health Sciences, University of Antwerp, Antwerp, Belgium; 5 Department of Otorhinolaryngology and Head and Neck Surgery, Antwerp University Hospital, Edegem, Belgium; 6 Department of Rehabilitation Sciences and Physiotherapy, Faculty of Medicine and Health Science, University of Antwerp, Antwerp, Belgium; 7 Multidisciplinary Motor Centre Antwerp (M2OCEAN), University of Antwerp, Antwerp, Belgium; 8 The Research Group MOVANT (MOVement ANTwerp), University of Antwerp, Antwerp, Belgium; 9 Department of Nutrition and Movement Sciences, NUTRIM School of Nutrition and Translational Research in Metabolism, Maastricht University Medical Centre+, Maastricht, The Netherlands; 10 Service of Otorhinolaryngology Head and Neck Surgery, Department of Clinical Neurosciences, Geneva University Hospitals, Geneva, Switzerland; 11 Maastricht University ENT Department, P. Debyelaan 25, 6229 HX Maastricht, The Netherlands

**Keywords:** Dynamic visual acuity, Bilateral vestibulopathy, Oscillopsia, Presbyvestibulopathy, Stabilization

## Abstract

**Introduction:**

Bilateral vestibulopathy (BVP) can affect visual acuity in dynamic conditions, like walking. This can be assessed by testing Dynamic Visual Acuity (DVA) on a treadmill at different walking speeds. Apart from BVP, age itself might influence DVA and the ability to complete the test. The objective of this study was to investigate whether DVA tested while walking, and the drop-out rate (the inability to complete all walking speeds of the test) are significantly influenced by age in BVP-patients and healthy subjects.

**Methods:**

Forty-four BVP-patients (20 male, mean age 59 years) and 63 healthy subjects (27 male, mean age 46 years) performed the DVA test on a treadmill at 0 (static condition), 2, 4 and 6 km/h (dynamic conditions). The dynamic visual acuity loss was calculated as the difference between visual acuity in the static condition and visual acuity in each walking condition. The dependency of the drop-out rate and dynamic visual acuity loss on BVP and age was investigated at all walking speeds, as well as the dependency of dynamic visual acuity loss on speed.

**Results:**

Age and BVP significantly increased the drop-out rate (*p* ≤ 0.038). A significantly higher dynamic visual acuity loss was found at all speeds in BVP-patients compared to healthy subjects (*p* < 0.001). Age showed no effect on dynamic visual acuity loss in both groups. In BVP-patients, increasing walking speeds resulted in higher dynamic visual acuity loss (*p* ≤ 0.036).

**Conclusion:**

DVA tested while walking on a treadmill, is one of the few “close to reality” functional outcome measures of vestibular function in the vertical plane. It is able to demonstrate significant loss of DVA in bilateral vestibulopathy patients. However, since bilateral vestibulopathy and age significantly increase the drop-out rate at faster walking speeds, it is recommended to use age-matched controls. Furthermore, it could be considered to use an individual “preferred” walking speed and to limit maximum walking speed in older subjects when testing DVA on a treadmill.

## Introduction

The vestibular system has two sets of peripheral organs that detect head accelerations and tilt. One of their main functions is to facilitate gaze stabilization. This is made possible to a large extent by a reflex from the vestibular organs to the eyes, called the vestibulo-ocular reflex (VOR) [1]. The VOR generates eye movements in the opposite direction of the head movements, stabilizing the eyes in space during head movements. As a result, the image of the environment remains stable on the retina while in movement.

In bilateral vestibulopathy (BVP), the vestibular function is reduced or absent on both sides [2]. This is a heterogeneous disorder in which the VOR, among other vestibular functions, is impaired [3]. It can lead to insufficient gaze stabilization, resulting in “blurred” vision during head movements, also known as “oscillopsia”. Oscillopsia is, next to imbalance, one of the main symptoms of BVP [2, 4–6]. BVP also has a strong negative impact on quality of life and social participation; many BVP-patients suffer from a constant psychological burden caused by fear of falling, decreased activity levels, and social isolation [2, 4, 5].

One way to quantify the functional outcome of gaze stabilization, is to test Dynamic Visual Acuity (DVA). DVA reflects the ability of the eyes to distinguish fine details in static objects during head movements [7]. DVA is often tested by comparing the visual acuity in a static condition (i.e., without head movements) to the visual acuity in a dynamic condition (i.e., with head movements). The loss of visual acuity in dynamic conditions (DVAL) is mostly used as outcome measure. Apart from being a functional outcome of the VOR, it also reflects the function of the visual, oculomotor, and vestibular system. That is why not all BVP-patients suffer from oscillopsia: other systems might compensate for the loss of VOR, such as otolith outputs [8, 9], automatic spinal locomotor programs [10], or compensatory walking strategies, e.g., reduction of walking speed or stride length [11]. The DVA can be tested in several ways, varying from passive head movements in an office chair, to walking on a treadmill [12–14]. For the latter, Guinand et al. [12] demonstrated a rise in test sensitivity for BVP with increasing locomotor speed: from 76% at 2 km/h to 97% when combining 2, 4, and 6 km/h.

It was previously reported that depending on the test protocol, up to 22% of BVP-patients were not able to complete the DVA test on a treadmill (drop-out), since they could not walk at the test speeds [12]. This increase in drop-out rate might have important implications when DVA while walking is considered as an outcome measure for therapeutic interventions, such as a vestibular implant [15]. However, age on its own, or in combination with BVP, has not yet been taken into account in previous studies, despite evidence of age related differences in gait variability and stability among healthy adults [16–19]. Furthermore, age might also influence DVA. For example, it has recently been demonstrated that DVA during self-generated side to side head movements remains stable in healthy individuals aged 3–49 years, but starts to decline at the age of 50 [20]. Therefore, age might significantly impact the feasibility of the DVA test on a treadmill. This might be important when investigating DVA in BVP, since BVP is more often seen at older ages: most patients are between 50 and 70 years [21].

The objective of this study was to investigate the effects of BVP and age when testing DVA on a treadmill. For this, DVA’s of BVP-patients and healthy subjects were evaluated on a treadmill at different speeds. It was hypothesized that BVP and age could significantly influence the drop-out rate and DVA, which might decrease the feasibility of the test in the BVP population.

## Methods

### Participants

Participants were recruited at a tertiary referral center (Maastricht UMC +) between June 2016 and December 2018. Inclusion criteria for BVP-patients were in accordance with the Diagnostic criteria Consensus document of the Classification Committee of the Bárány Society [22]: a horizontal angular VOR gain on both sides < 0.6 (angular head velocity 150–300°/s) and/or summated slow phase velocity of nystagmus of less than 6°/s on each side during bithermal caloric tests (30 and 44 °C, 300 ml in 30 s) and/or a horizontal angular VOR gain < 0.1 upon sinusoidal stimulation on a rotatory chair (0.1 Hz, Vmax = 50°/s) and/or a phase lead > 68° (time constant of < 5 s). In addition, patients needed to be older than 18 years. Patients unable to stop medication against anxiety or depression 1 week before testing, were excluded from the study, as well as those suffering from peripheral neuropathy of the lower extremities.

Healthy subjects were recruited via posters in the hospital and among families and friends of the researchers. A questionnaire was used to rule out, as much as possible, any deficits or diseases that could influence the vestibular system. It comprised the following topics: previous medical history (including otorhinolaryngological, neurological, ophthalmological); use of any medication; known balance problems, recent neck trauma or dizziness in the past 6 months.

All participants were excluded from the study if they were unable to walk on the treadmill at 2 km/h or had a vision of − 4.0 Diopter or lower (without correction), in which they could not read the first line of the optotypes on the computer screen. In some cases, BVP-patients were allowed to hold handrails of the treadmill to prevent falling (10 BVP-patients). The use of alcohol or other stimulants was forbidden in the 24 h before examination.

### Evaluating age effect on the vestibular function in BVP-patients

The potential effect of age on vestibular function in BVP-patients (which could affect drop-out rate and DVA) was assessed using outcomes from two tests: the video Head Impulse Test (vHIT, ICS Impulse, GN Otometrics; Taastrup, Denmark) and the caloric test using bithermal (30° and 40° C) irrigations of water. vHIT gains were calculated for the leftward and rightward directions in the lateral plane, and for the upward and downward directions in right anterior—left posterior and left anterior—right posterior planes. The sum of bithermal maximum peak slow phase velocities (SPV) of the nystagmus was used as outcome measure of the caloric test, calculated separately for each side.

### Testing DVA on a treadmill

Sloan optotypes (C, D, H, K, N, O, R, S, V and Z) projected on a computer screen were used to test visual acuity. The computer screen was placed at eye level and at 2.8 m from the subject. A Sloan letter was presented on a computer screen (LG 24bk55 24″), using a custom program written in Matlab R2010a (The Mathworks, Natick, MA, USA). The program randomly showed five letters in a single-letter sequence at one logarithm of the Minimum Angle of Resolution (logMAR). If a minimum of two out of five letters were correctly recognized, the letter size decreased by 0.1 logMAR and five new letters were shown, one after another. If less than two out of five letters were recognized, the procedure was stopped.

The visual acuity of all BVP-patients and healthy subjects was tested in two conditions: static and dynamic. Static visual acuity was measured when the subject was standing still on the treadmill (1210 model, SportsArt, Inc., Tainan, Taiwan). Visual acuity in dynamic conditions was measured while walking on the treadmill at different speeds (2, 4, 6 km/h, non-randomized). The study procedure ended either when all walking speeds were completed, or when subjects could not walk at a higher speed. If subjects were not able to complete the test at a specific walking speed, they were considered as a “drop-out” for that walking speed. To ensure the subject’s safety, a safety string was clipped to the subject’s waist that was connected to the emergency brake of the treadmill.

### Data analysis and statistics

Visual acuity in static and dynamic conditions was calculated as: LogMAR = 0.1 + LogMAR_*x*_ − 0.02*y* [23], where “*x*” was defined as the last optotype line in which two or more letters were read correctly and “*y*” was defined as the number of correctly read letters at that line.

DVAL was defined as the difference between visual acuity in the static condition and the dynamic conditions. Note that a negative DVAL indicates poorer vision in the tested dynamic condition compared to the static condition. Descriptive statistics were made for age and DVAL. The independent sample *t* test was used to compare mean age between groups.

To evaluate whether holding the treadmill handrails affected DVAL, an independent *t* test was used to assess whether a difference in mean DVAL existed between patients who held the treadmill handrails and patients who did not hold the treadmill handrails. Obtained *p* values were Bonferroni corrected.

The potential effect of age on vestibular function in BVP-patients was analyzed using linear regression analyses. Each model contained age as an independent variable and the corresponding outcome (gain or SPV) as a dependent variable.

Since drop-out at the speed of 4 and 6 km/h was perfectly correlated (e.g., drop-out at 4 km/h excluded successful completion of the test at 6 km/h), multilevel logistic regression of drop-out (yes or no) on speed, age and group (BVP-patients or healthy subjects) failed (multicollinearity). Therefore, a new dependent variable reflecting the missing pattern was formed using the following criteria: pattern 1—no drop-out at all speeds, pattern 2—drop-out at 6 km/h, and pattern 3—drop-out at 4 and 6 km/h. Then multinomial logistic regression was performed to determine the dependency of drop-out on group and age.

To analyze the effect of age and speed on DVAL, while accounting for the dependence among measurements of the same participant, a linear-mixed effects model was applied. Initially, age, group, speed, and all their two-way interactions were included as fixed factors. Then, the non-significant interactions were removed by backward selection. Finally, age, group, speed and group by speed interaction were left in the model. Pairwise comparisons were made per group to compare DVAL at 2, 4, and 6 km/h. Pairwise comparisons were also made per speed to compare DVAL in BVP-patients and healthy subjects. The significance level, alpha, was set to 0.05. In case of multiple comparisons, *p* values were Bonferroni corrected. Data were analyzed in R (v.3.5.2) and SPSS (v.25).

### Ethical considerations

This study was in accordance with the Declaration of Helsinki (amended version 2013) Approval was obtained by the ethical committees of Maastricht University Medical Centre (NL52768.068.15/METC 151027). All participants provided written informed consent prior to the study.

## Results

### Participants

Forty-four BVP-patients (20 male, mean age 59 years, standard deviation 11 years) and 63 healthy subjects (27 male, mean age 46 years, standard deviation 20 years) were included in this study. Although mean age was significantly higher in BVP-patients (*p* < 0.001), ages of 60% of the healthy subjects were equally distributed within the age range of the tested BVP-patients (41–83 years). Age characteristics of both groups are presented in Table 1. Etiologies of BVP comprised: gentamicin treatment (*n* = 5), vancomycin (*n* = 1), amikacin (*n* = 1), chemotherapy (*n* = 1), Herpes infection (*n* = 1), meningitis (*n* = 3), Hashimoto’s disease (*n* = 1), renal failure (*n* = 1), Meniere’s Disease (*n* = 4), sequential acute unilateral vestibulopathy (*n* = 1), and genetic (*n* = 5). The etiology remained idiopathic in 20 subjects.

**Table 1:** Age characteristics of the tested groups. The number of participants is shown per age group for both BVP-patients and healthy subjects

	Age group (years)					
	19–30	31–40	41–50	51–60	61–70	71–83
BVP-patients	1	0	9	14	16	4
Healthy subjects	23	2	10	10	10	8

In BVP-patients no significant age effect was found on the outcomes of the vHIT and caloric test (*p* ≥ 0.161).

### Drop-out

**Fig. 1:**
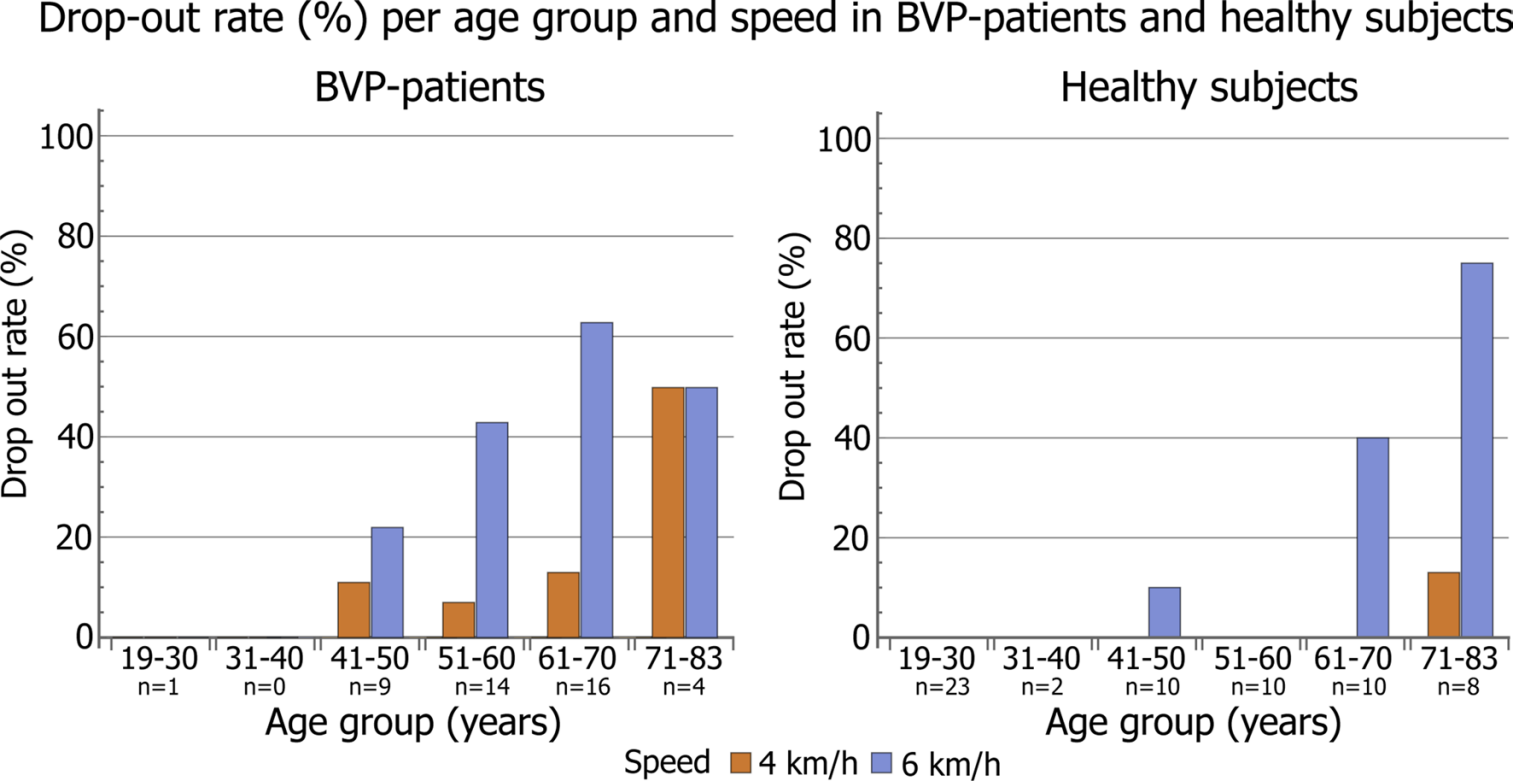
Drop-out rate (%) in BVP-patients (left) and healthy subjects (right) per age group and speed

Not all BVP-patients and healthy subjects were able to complete the DVA test at each walking speed, which resulted in dropping-out. The drop-out rate per group, walking speed, and age decade is presented in Fig. 1. Age significantly increased the odds of dropping-out at 6 km/h (odds ratio = 1.12, *p* < 0.001), and both age and BVP increased the odds of dropping-out at 4 km/h, and consequently at 6 km/h (Age: odds ratio = 1.15, *p* = 0.008; BVP: odds ratio = 12.40, *p* = 0.038).

### Dynamic visual acuity loss

**Fig. 2:**
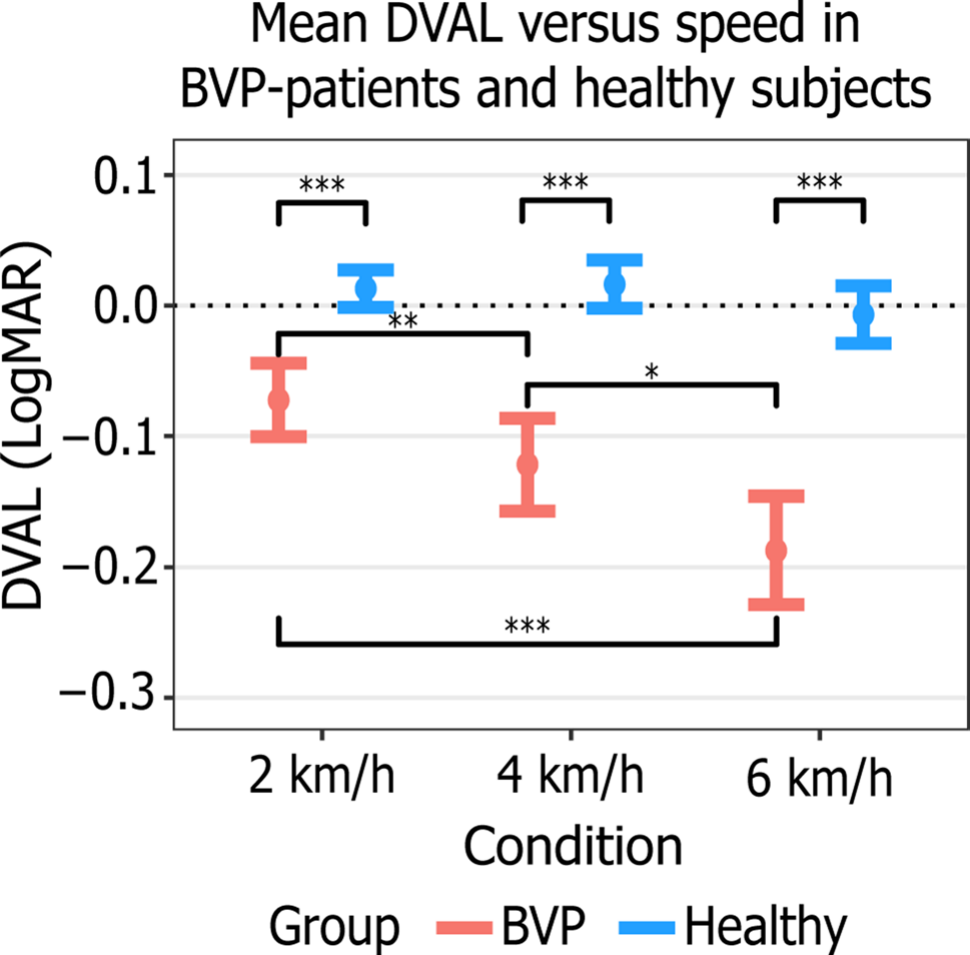
Mean DVAL versus speed in BVP-patients (red) and healthy subjects (blue). DVAL was calculated as the decline in logMAR between VA_static_ and VA_dynamic_. Therefore, a negative DVAL value indicates poorer vision while walking on the treadmill, compared to standing still. Error bars represent 95% confidence interval. Three asterisks (***) indicate *p* < 0.001, two asterisks (**) indicate *p* < 0.01, one asterisk (*) indicates *p* < 0.05

DVAL was significantly lower in BVP-patients at all walking speeds (*p* < 0.001) (Fig. 2). Neither age (*p* = 0.399) nor speed (*p* ≥ 0.258) had a significant effect on DVAL in the group of healthy subjects. In the group of BVP-patients only speed (*p* ≤ 0.036) significantly influenced DVAL: it decreased with an increase of speed. There were no significant differences in DVAL across speeds in patients who did and did not hold the treadmill handrails (*p* ≥ 0.29).

## Discussion

This study investigated the drop-out rate and DVA of BVP-patients and healthy subjects. BVP and age were hypothesized to significantly influence the drop-out rate and DVA obtained in both groups. It was demonstrated that both BVP and age, significantly increased the drop-out rate in both groups at the speed of 4 km/h or higher. Regarding DVA, only BVP (not age), significantly decreased DVA in the subjects who were able to walk on the treadmill. Furthermore, DVA significantly decreased with higher walking speeds only in BVP patients. To our knowledge, this study has the largest group of BVP patients in which the influence of BVP and age on the drop-out rate and DVA was tested on a treadmill [12, 14].

A higher drop-out rate in both BVP patients and healthy subjects with increasing age and walking speed could be explained by an age-related multisensory decline of the systems involved in maintaining posture and gait [24–26]. In the group of healthy subjects, this might also include presbyvestibulopathy: an age-related decline of the vestibular function [27]. Since BVP mostly occurs at older ages, and BVP-patients are often unable to walk at speeds higher than 5 km/h [21, 26, 28], it might be questioned whether testing DVA at fixed speeds on a treadmill would be a reasonable outcome measure for vestibular rehabilitation of BVP-patients in a research setting. However, testing DVA while walking is only one of the few “close to reality” functional outcome measures of vestibular function in the vertical plane [12]. It could, therefore, be proposed to individually adjust the walking speed for each patient, using their “preferred” walking speed. In older subjects on group level, the preferred walking speed range would probably be between 2 km/h and (below) 6 km/h. After all, mean DVA is already significantly reduced at 2 km/h [12], the self-selected walking speed for patients with vestibular dysfunction is about 3 km/h [29, 30], and at 6 km/h both healthy subjects and BVP patients above 40 years show a significantly increased drop-out rate. The use of a preferred walking speed was already successfully demonstrated in BVP-patients fitted with a prototype vestibular implant [31]. Nevertheless, other possible functional outcome measures could also be hypothesized which do not involve walking. For instance, testing DVA with actively generated head movements while sitting on a chair [20] or the functional head impulse test [32]. However, it should be taken into account that each specific way of testing DVA examines (to a certain extent) different parts, sensitivities and mechanisms of the vestibular system: e.g., the semicircular canals, otolith organs, low- and high-frequency sensitivity, and compensatory strategies. This has already been revealed by the lack of correlation between outcomes of the DVA test on a treadmill, and the functional head impulse test [33].

Conflicting evidence exists regarding the effect of age on DVA when tested on a treadmill in healthy subjects. One previous study described a significant age effect on DVA at only 4 km/h (not at 3, 6, and 9 km/h) [14], while another study found a significant age effect at 4 and 6 km/h [12]. The present study did not find any effect of age on DVA. This might partially be explained by different inclusion criteria used for the group of healthy subjects; Verbecque et al. [14] used a questionnaire and age-specific static balance testing, Guinand et al. [12] used the Video Head Impulse Test, and this study used a questionnaire to rule out (as much as possible) any deficits or diseases that could influence the vestibular system. Furthermore, the different statistical analyses and testing paradigms, including the used optotype charts and DVA cut-off values, could contribute to the conflicting evidence. Nevertheless, the findings of this study do not rule out any age effect, since the DVA in the group of subjects who dropped-out, remains unknown. Furthermore, DVA tested during self-generated side to side head movements significantly declines from the age of 50 [20]. Therefore, taking the drop-out rate and the evidence regarding age effect into account, it would be recommended to use age-matched controls when testing DVA in research settings and to limit maximum walking speed for older subjects (e.g., below 6 km/h).

BVP-patients showed, on group level, worse DVA than healthy subjects at all walking speeds. This finding is congruent with previously described results [12]. In addition to impaired vestibular function, this DVA decrease might (partially) be induced by attention deficits [34] or the inability to correctly perform dual-tasks, which can be present in BVP-patients [35]. However, DVA overlapped between BVP-patients and age-matched controls. This can be explained by multiple factors. First, the DVA is a functional outcome of a multisensory system. Input from the visual, vestibular and oculomotor systems is centrally processed, which facilitates adaptation and compensation mechanisms. An example of such a compensation mechanism is minimizing head movements to improve gaze stabilization. Second, DVA can be trained. It has been shown that vestibular rehabilitation exercises facilitate the recovery of gaze during head movements in BVP-patients [36]. Furthermore, in patients with unilateral peripheral vestibulopathy, covert saccades can improve DVA [37, 38]. Third, during the DVA test on a treadmill, (partially) active movements are made. These active movements are less useful in discriminating between BVP-patients and healthy subjects, as compared to passive movements [13, 39]. In addition, during stereotyped locomotion, feed-forward signals from an efference copy of the locomotor commands suppress the VOR in the vertical plane, which can mediate gaze stabilization [40]. Finally, walking speed affects gait parameters in BVP-patients [26]. All these factors imply that the DVA test on a treadmill should mainly be used to evaluate the functional status of the vestibular system.

### Limitations of the study

In contrast to previous studies [12, 14] only a questionnaire was used to determine whether a subject was healthy or not. None of the vestibular tests like the Video Head Impulse Test or caloric test were performed in healthy subjects. It cannot be ruled out that results of the healthy subjects might have been influenced by factors like presbyvestibulopathy or asymptomatic vestibulopathies. This could mainly imply that the effects of BVP on drop-out and DVA found in this study, might be underestimated. Height of the participants was not measured, which could affect stride length and, therefore, head movement amplitude and, consequently, DVA. Furthermore, statistical power in the highest age groups was small due to the high drop-out rate. It can, therefore, only be stated that DVA was not significantly influenced by age in BVP-patients and healthy subjects who were able to walk at the tested speeds. The DVA of subjects who were unable to walk could not be determined using the treadmill test.

## Conclusion

DVA tested while walking on a treadmill, is one of the few “close to reality” functional outcome measures of vestibular function in the vertical plane. It is able to demonstrate significant loss of DVA in bilateral vestibulopathy patients. However, since bilateral vestibulopathy and age significantly increase the drop-out rate at faster walking speeds, it is recommended to use age-matched controls. Furthermore, it could be considered to use an individual “preferred” walking speed and to limit maximum walking speed in older subjects when testing DVA on a treadmill.

## Data Availability

Data is available on the reasonable request to the corresponding author.
